# Physical activity behavior pathway from intention to action in older adults: a structural equation model based on the multi-process action control framework

**DOI:** 10.3389/fpsyg.2026.1786169

**Published:** 2026-07-01

**Authors:** Fan Liu, Yuxuan Zhang, Jinjin Yuan, Yingdan Chen, Kexing Si, Ruirui Zhang, Xiuxiu Huang, Bingqian Zhu

**Affiliations:** 1 School of Nursing, Shanghai Jiao Tong University, Shanghai, China; 2 School of Exercise and Health, Shanghai University of Sport, Shanghai, China; 3 School of Nursing, University of North Carolina at Chapel Hill, Chapel Hill, NC, United States; 4 Department of Nursing, Shanghai Jiao Tong University School of Medicine Affiliated Renji Hospital, Shanghai, China

**Keywords:** intention-action gap, older adults, physical activity, structural equation model, the multi-process action control framework

## Abstract

**Objectives:**

This study aimed to examine the behavioral pathway from intention formation to action control of physical activity, and thereby provide empirical evidence for developing physical activity promotion programs among older adults.

**Methods:**

Older adults were recruited from community health centers in Shanghai, China. Physical activity was assessed by the Physical Activity Scale for the Elderly. Physical activity intention was measured by a single validated item. Variables associated with intention formation and action control for physical activity were collected using standardized tools based on the multi-process action control framework. Structural equation modeling analysis was performed to examine the potential path relations.

**Results:**

A total of 372 older adults (mean age 73.2) were included. Among them, 89.2% (*n* = 332) reported an intention to be physically active and only 18.1% (*n* = 60) of these intenders met the WHO physical activity recommendations. At the intention formation stage, perceived opportunity was the only factor directly associated with intention. At the action adoption stage, perceived opportunity, affective attitude, and intention were all directly associated with behavioral regulation. Furthermore, intention partially mediated the relation between perceived opportunity and behavioral regulation. At the action maintenance stage, behavioral regulation indirectly influenced leisure-time physical activity through habit and identity.

**Conclusion:**

Perceived opportunity is key to physical activity intention, and together with affective attitude, it contributes to promoting intention translation. Habit formation and identity affirmation are vital to maintaining adequate physical activity among older adults.

## 1 Introduction

Promoting healthy aging has become a global priority with the acceleration of population aging. Adequate physical activity is critical for healthy aging given its role in achieving optimal physical and mental function (Sempere-Rubio et al., 2023). Older adults are recommended to engage in at least 150 min of moderate-intensity or 75 min of vigorous-intensity aerobic physical activity per week (Piercy et al., 2018). However, only about one-fifth meet the recommendation (Hakimi et al., 2024), despite most having the intention to do so, implying a significant intention-action gap. Currently, physical activity intention and action are typically examined separately by different studies, which cannot directly reflect the actual intention-action gap in older adults. A recent meta-analysis integrated the intention and action profiles among students and adults, and revealed an intention-action gap of 47.6% (Feil et al., 2023). Evidence from older adults remains scarce. Given the urgent need to promote physical activity among older adults, it is imperative to investigate their intention and action profiles and uncover the underlying pathway from intention formation to action.

Recently, researchers have started to pay attention to the process of intention formation to action control in the area of physical activity. Zhang et al. (2019) investigated adults’ intention to walk and their actual walking behavior in the workplace. It was found that attitude, subjective norm, and perceived behavioral control indirectly influenced walking behavior through intention. Another study (Xin et al., 2019) further demonstrated that habit played a significant mediating role between intention and action. Although the above studies provided promising results, there was a lack of comprehensive exploration of factors involved in different stages from intention formation to action control. More importantly, the underlying pathway was not identified. In addition, prior studies have focused primarily on younger or middle-aged adults. They usually have different scheduling, physical activity goals, and physical function from older adults. Therefore, investigations among older adults are needed.

In 2016, Rhodes (2017) proposed the Multi-Process Action Control (M-PAC) framework to explore the potential behavioral pathway for physical activity. Based on this framework, physical activity involves three key processes. First, intention formation is grounded in reflective processes, including instrumental attitude (outcome expectations), affective attitude (emotional experiences), perceived capability, and perceived opportunity. Second, action adoption is a crucial mediator linking intention with action. It is dependent on behavioral regulation processes and is influenced by affective attitude (the proximal affective experiences following a behavior) and perceived opportunity. Third, action maintenance is closely associated with reflexive processes such as habit (automaticity) and identity (self-categorization). It is important to note that this framework was specifically designed for autonomous exercise. Recently, it has been has been applied to explain the underlying pathway from intention to action across different populations, such as patients with cancer (Vallerand et al., 2016) and parents (Rhodes et al., 2021). Using the M-PAC framework, Tabaczynski et al. (2023) found that affective attitude and perceived capability correlated with individuals’ intention, whereas identity was associated with actual physical activity behavior among cancer patients. Grant et al. (2022) reported that perceived capability, habit, and identity could predict physical activity behavior among mothers with young children. Among Korean university students (Lee et al., 2025), affective attitude, perceived opportunity, and perceived capability were significant predictors of intention. Intention further predicted physical activity through the sequential mediation of behavioral regulation, followed by identity and habit, highlighting the distinct pathways from intention formation to sustained action. Previous evidence showed that different pathways in the M-PAC framework might work in various populations. It warrants further investigation into which pathways played important roles in shaping physical activity behaviors among older adults.

To the best of our knowledge, the M-PAC framework has not been used to examine the intention-action process of physical activity among older adults. Older adults face distinct age-related shifts that may alter the intention-action process. Unlike younger cohorts, whose physical activity is often driven by motivation, older adults’ translation of intention into action is frequently disrupted by physical vulnerability (Chruścińska et al., 2026). They often exhibit a “ceiling effect” in exercise intention (strong desire to be healthy) but struggle with execution due to a heightened fear of injury and functional limitations (Chen et al., 2023). Therefore, evidence from younger or healthier groups may not generalize to the older population. Guided by the M-PAC framework and informed by the above empirical evidence, this study hypothesized that instrumental attitude, affective attitude, perceived capability and perceived opportunity were positively associated with physical activity intention. Second, behavioral regulation was proposed as a crucial mediator linking intention with physical activity maintenance. Furthermore, we assumed that affective attitude and perceived capability would exert direct effects on behavioral regulation, independent of intention. Finally, we hypothesized that habit and identity would partially mediate the influences of behavioral regulation on the maintenance of physical activity. This study would reveal how older adults translate their physical activity intention into action via structural equation modeling, and provide valuable references for developing effective interventions to promote physical activity.

## 2 Materials and methods

### 2.1 Study design

The present study adopted a cross-sectional design and was reported according to the Strengthening the Reporting of Observational Studies in Epidemiology (STROBE) checklist (Cuschieri, 2019). Ethical approval was obtained from the Ethics committee of Shanghai University of Sport (Approval Number: 102772025RT016).

### 2.2 Hypothetical model

**Figure 1:**
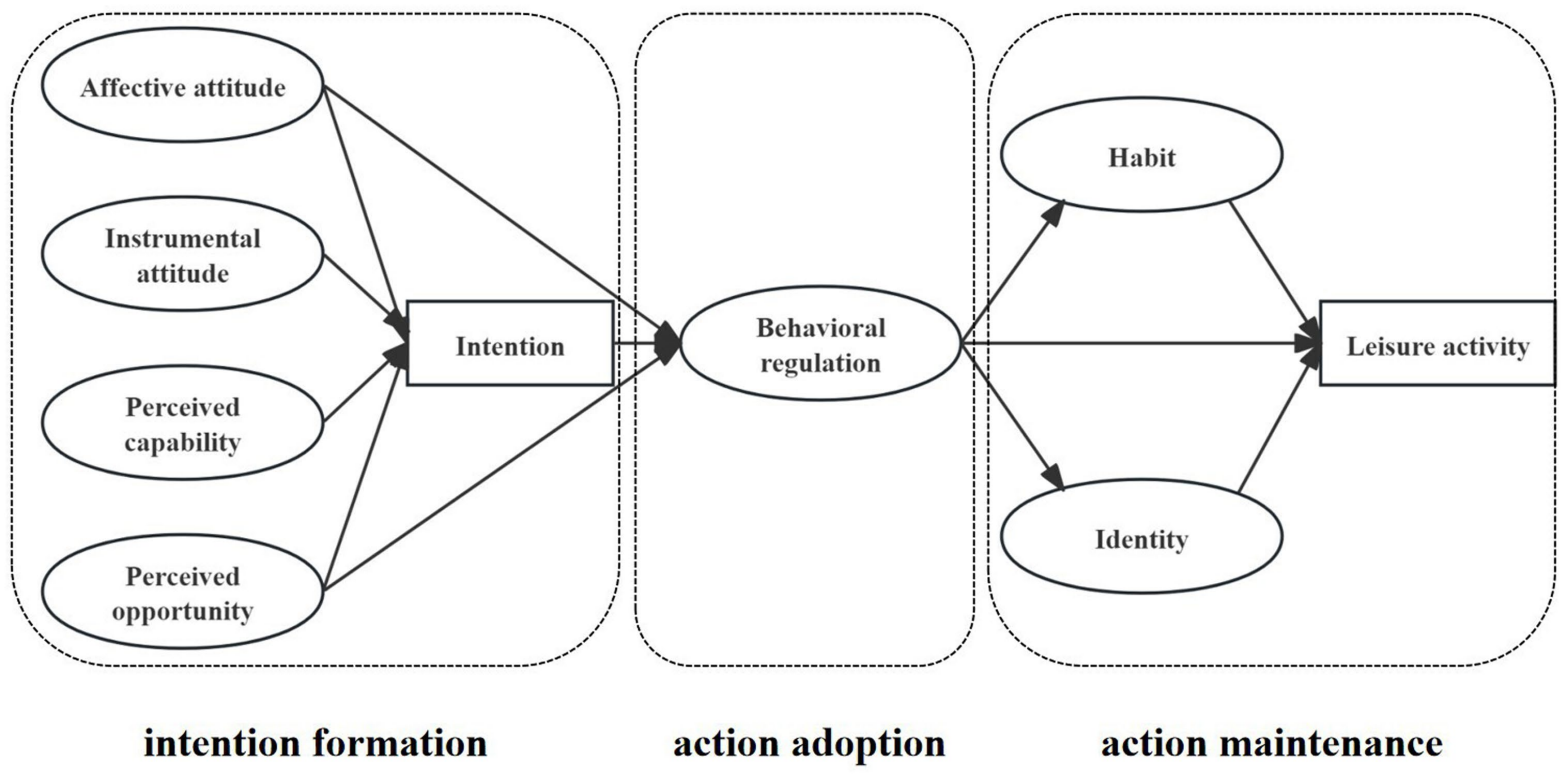
Theoretical model and hypotheses.

We proposed a hypothesized model to depict the path relations among the relevant variables, and elucidate how older adults translate intention into action based on literature review and the M-PAC framework. It incorporated three stages of physical activity. The detailed hypotheses are listed in Figure 1.

### 2.3 Participants

Participants were recruited from community health service centers in Shanghai, China. The inclusion criteria were: (1) aged 60 years or older; (2) living in communities; (3) able to independently perform basic activities of daily living, with the Barthel index score≥60 (Wade and Collin, 1988); (4) gave informed consent. Individuals were excluded if they: (1) were participating in another research project related to physical activity; or (2) had acute cardiopulmonary dysfunction or unstable clinical conditions over the past 6 months, considering that the investigation could not reflect their real physical activity. Previous literature suggested that the sample size for structured equation modeling should be at least 10 times the number of variables (Hair, 2009). Our study had 27 measurable independent variables, requiring at least 270 participants. Taking into account 20% of invalid samples (Wang and Ji, 2020), a total of 338 participants was deemed sufficient for this study. Written informed consent was obtained from all participants prior to data collection. Face-to-face surveys were conducted in a quiet, private room by investigators who have received unified training. All participants completed the survey under the one-on-one guidance of investigators via an electronic data collection platform (H6WORLD) developed by Peking University, which has been used in previous research (Shen et al., 2024). Detailed information about the collected data was as follows.

### 2.4 Measurements

We measured participants’ demographic information, physical activity intention, and physical activity levels. According to the M-PAC framework, we also collected potential factors associated with intention formation, action adoption, and action maintenance. All questionnaires were Chinese versions. Detailed measures were as follows.

#### 2.4.1 Demographic information

Demographic information was collected, including age, gender, education, marital status, and monthly income. Self-reported height and weight were also collected to calculate body mass index.

#### 2.4.2 Physical activity intention

Physical activity intention was measured by a single continuous open-ended item (*How many days per week, on average, do you plan to exercise over the next three months*) (Rhodes and Rebar, 2017), which was developed by Courneya (1994). Participants could be categorized as intenders (≥3 days per week) or non-intenders (<3 days per week) (Tabaczynski et al., 2023). This measurement has been widely used and showed high reliability and flexibility (Rhodes et al., 2004).

#### 2.4.3 Physical activity behavior

Physical activity behavior was measured using the Chinese version of Physical Activity Scale for the Elderly (Washburn et al., 1993), which demonstrated good reliability and validity among older adults (Sattler et al., 2020). It consists of three subscales to assess leisure-time physical activity, household activities, and work-related activities, respectively. In this study, we mainly focused on the subscale of leisure-time physical activity given that the M-PAC framework focuses on autonomous exercise, rather than mandatory activities, such as housework and work-related activities. Leisure-time physical activity includes walking, low-intensity, moderate-intensity, and high-intensity activities, as well as resistance training. The scale assesses the frequency and average duration per session of each activity over a one-week period to calculate the total weekly time spent on each type. Meeting the equivalent of at least 150 min per week of moderate- to vigorous-intensity physical activity was used as the criterion for determining the presence of exercise behavior (Piercy et al., 2018). A leisure-time physical activity score was computed by multiplying the activity-specific weight by the total weekly duration of each activity and summing the results (Li et al., 2023). This score was then used as the outcome variable in a structural equation model to examine the intention-action transition pathway within the M-PAC framework.

#### 2.4.4 Variables related to intention-action process

##### 2.4.4.1 Variables associated with intention formation

Affective attitude and instrumental attitude were measured using two separate 3-item scales developed by Rhodes and Courneya (2003). Each item is rated on a 7-point Likert scale, with a higher score indicating a more positive affective or instrumental attitude towards physical activity. Similarly, perceived capability and perceived opportunity were assessed by two separate 3-item scales developed by Rhodes et al. (2006) and Williams and Rhodes (2016), respectively. Each item is also rated on a 7-point Likert scale. A higher score indicates a higher perception of capability and opportunity. Both of the measurements has been used previously (Sui et al., 2024).

##### 2.4.4.2 Variables associated with action adoption

Behavioral regulation was assessed using the Action Planning Questionnaire, developed by Sniehotta et al. (2005). It consists of six items, with each item scored on a 5-point Likert scale. A higher score indicates a greater degree of self-regulation. The scale has been used in older adults and showed good reliability and validity (Sniehotta et al., 2006).

##### 2.4.4.3 Variables associated with action maintenance

Habit was measured by the Self-Report Behavioral Automaticity Index (Gardner et al., 2012). This tool has four items to assess the automaticity of physical activity. Identity was assessed using the Exercise Identity Scale (Anderson and Cychosz, 1994). It measures four aspects, including self-evaluation, other-evaluation, attitudes towards regular physical activity, and self-description. For both tools, participants indicated the degree to which they agreed with each statement on a 5-point Likert scale. They have been used in older adults and showed good reliability and validity (Palermo and Rancourt, 2023).

### 2.5 Data analyses

Data were analyzed using SPSS 27.0 (IBM., Armonk, N. Y., USA) and AMOS 28.0 (IBM., Armonk, N. Y., USA). Missing patterns were assessed using Little’s MCAR test. Listwise deletion was used if missing completely at random (Little, 1988). Individuals who planned to exercise for three or more days in the upcoming week were classified as intenders, while achieving at least 150 min of weekly moderate-to-vigorous physical activity served as the benchmark for successful action. Based on the combination of these two criteria, participants were categorized into four distinct profiles: Successful Intenders, Unsuccessful Intenders, Non-intenders without action, and Non-intenders with action. The intention-action gap was calculated by dividing the number of unsuccessful intenders by the total number of intenders (Rhodes et al., 2008). Continuous variables were first assessed for normality using the Shapiro–Wilk test. Variables following a normal distribution were expressed as mean ± standard deviation (SD) while non-normally distributed variables were reported as median (interquartile range, IQR). Pearson correlation was used for data with normal distribution, Spearman correlation was used otherwise. Categorical variables were described using frequencies and percentages. Structural equation modeling was used to test the hypotheses proposed in Figure 1. For data with a non-normal distribution, the bias-corrected percentile bootstrap method (5,000 iterations) was used. This approach can provide a more rigorous test of direct and indirect effects in the presence of non-normality (Cheung and Lau, 2008). This procedure ensures more reliable standard errors, confidence intervals, and parameter estimates, providing a more rigorous test of direct and indirect effects in the presence of non-normality. To ensure the rigor of the measurement model, we evaluated the reliability and convergent validity of the scales using Composite Reliability (CR) and Average Variance Extracted (AVE). The criteria for evaluating goodness-of-fit were as follows: χ^2^/df value < 5.00, comparative fit index (CFI) > 0.90, normed fit index (NFI) > 0.90, Tucker-Lewis index (TLI) > 0.90, Bollen’s incremental fit index (IFI) > 0.90, and root mean square error of approximation (RMSEA) < 0.08 (Kline, 2016). To assess the robustness of the findings, we performed a sensitivity analysis using multiple imputation under the missing completely at random assumption. The *p* value less than 0.05 was considered statistically significant.

## 3 Results

### 3.1 Participant characteristics

A total of 397 older adults were recruited, and 25 missing cases were excluded due to missing completely at random. Thus, 372 were included in the final analyses, with a non-response rate of 6.72%. The average age of the participants was 73.21 (SD = 5.31, range = 61–95) years old, and 230 (61.8%) participants were women. The mean scores were 5.92 (SD = 2.07) for physical activity intention and 27.01 (SD = 24.04) for leisure-time physical activity, respectively. Detailed information is listed in Table 1.

**Table 1:** Demographic characteristics of the participants (*n* = 372).

Variables	*N* (%)/Mean(SD) Median [IQR]	Range
Gender		
Women	230 (61.8)	
Man	142 (38.2)	
Education		
Middle school and below	181 (48.7)	
High school and above	191 (51.3)	
Marital status		
Married	312 (83.9)	
Single/widowed	60 (16.1)	
Monthly income (CNY)^a^		
≤4,000	59 (15.9)	
4,000–7,000	272 (73.1)	
>7,000	41 (11.0)	
Comorbidity		
Yes	159 (42.7)	
No	213 (57.3)	
Age (years)	73.21 (5.31)	61–93
Body mass index (kg/m^2^)	23.50 (3.28)	15.40–42.46
Leisure-time physical activity	25.71[8.57, 34.71]	0–207.22
Affective attitude	6[5, 6]	1–7
Instrumental attitude	7[6, 7]	1–7
Perceived capability	5.5[3, 7]	1–7
Perceived opportunity	5[4, 6]	1–7
Intention	7[4, 7]	0–7
Behavioral regulation	2[1, 3]	1–5
Habit	4[3, 4.75]	1–5
Identity	3.75[2.5, 4]	1–5

^a^CNY, Chinese Yuan.

### 3.2 Participants’ intention and action profiles

**Figure 2:**
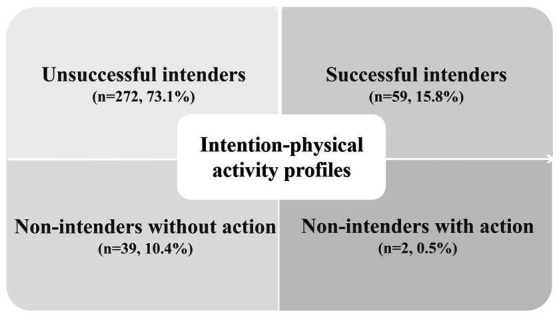
Intention-physical activity profiles. Successful intenders: participants with physical activity intention and action; Unsuccessful intenders: participants with physical activity intention but without action; Non-intenders with action: participants with physical activity action but no intention; Non-intenders without action: participants without physical activity action and intention.

Among them, 332 (89.2%) were identified as intenders, with the remaining 40 (10.8%) having insufficient intention. Among the intenders, only 60 (18.1%) achieved the recommended physical activity level and were identified as successful intenders. The intention-action gap was 81.9%. Detailed intention-action profiles are displayed in Figure 2.

### 3.3 Bivariate associations

Table 2 shows the bivariate associations between variables of interest. The results indicated that all variables exhibited significant positive correlations. The variance inflation factor values ranged from 1.32 to 1.83, suggesting no severe multicollinearity among the variables.

**Table 2:** Bivariate associations between variables of interest (Spearman).

Variables	1	2	3	4	5	6	7	8	9
1 Affective attitude	—								
2 Instrumental attitude	0.531**	—							
3 Perceived capability	0.391**	0.241**	—						
4 Perceived opportunity	0.322**	0.303**	0.492**	—					
5 Behavioral regulation	0.226**	0.177**	0.244**	0.239**	—				
6 Habit	0.353**	0.330**	0.292**	0.372**	0.171**	—			
7 Identify	0.405**	0.406**	0.263**	0.362**	0.481**	0.466**	—		
8 Intention	0.194**	0.168**	0.138**	0.287**	0.307**	0.400**	0.377**	—	
9 Leisure-time physical activity	0.229**	0.232**	0.235**	0.245**	0.201**	0.291**	0.345**	0.238**	—

**p < 0.01.

### 3.4 Structural equation modelling results

**Figure 3:**
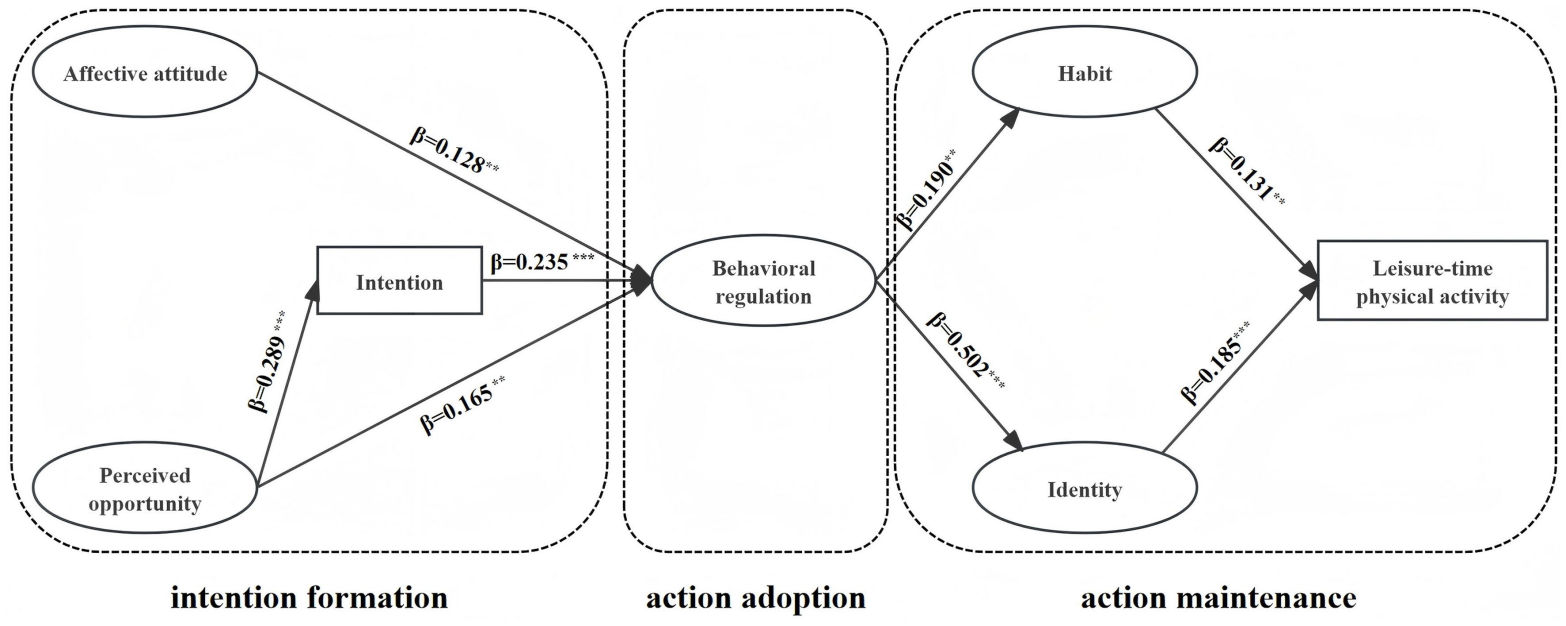
Final model with standardized model paths. **p < 0.01; ***p < 0.001.

We first examined the hypothesis model and found several non-significant path relations (Supplementary Table S1). In particular, affective attitude, instrumental attitude, and perceived capability were not associated with intention. The direct path relation between behavioral regulation and leisure-time physical activity was not significant. A chi-square difference test was performed to compare the initial model with a nested model in which all non-significant paths were fixed to zero. The test was non-significant (Δx^2^ = 7.49, *p* = 0.112), supporting the removal of these paths. Consequently, the two isolated latent variables were removed for parsimony, resulting in the final model (Figure 3).

The model showed a satisfactory model fit, with χ^2^/df = 3.35, RMSEA = 0.08, TLI = 0.93, NFI = 0.93, IFI = 0.95, and CFI = 0.95. All factor loadings and path coefficients were statistically significant (*p* < 0.05). As illustrated in Supplementary Table S2, all latent constructs demonstrated robust psychometric properties. AVE for each dimension exceeded the 0.50 threshold (ranging from 0.698 to 0.991), and CR values were all above 0.70 (ranging from 0.895 to 0.997). Perceived opportunity was the only variable associated with intention (*β* = 0.289, 95% CI [0.164 to 0.413], *p* < 0.001) during the intention formation process. Perceived opportunity (*β* = 0.165, 95%CI [0.059, 0.278], *p* = 0.003), affective attitude (*β* = 0.128, 95% CI [0.037, 0.210], *p =* 0.006) and intention (*β* = 0.235, 95% CI [0.138, 0.321], *p* < 0.001) were directly positively correlated with behavioral regulation. Intention partially mediated the association between perceived opportunity and behavioral regulation (*β* = 0.039, 95% CI [0.020, 0.070], *p* < 0.001). During the process of action maintenance, behavioral regulation was indirectly associated with leisure-time physical activity through habit (*β* = 0.602, 95% CI [0.158, 1.433], *p* = 0.003) and identity (*β* = 2.252, 95% CI [1.040, 3.836], *p* < 0.001). Sensitivity analyses (Supplementary Table S3) confirmed that the model was highly robust. The detailed effect size for each path is listed in Figure 3 and Table 3.

**Table 3:** Standardized direct and indirect pathways of the model.

Paths	β	SE	BC (95% CI)		*p*
			Lower	Upper	
Intention formation					
Perceived opportunity → Intention	0.289	0.065	0.164	0.413	<0.001
Action adoption					
Affective attitude → Behavioral regulation	0.128	0.044	0.037	0.210	0.006
Perceived opportunity → Behavioral regulation	0.165	0.055	0.059	0.278	0.003
Intention → Behavioral regulation	0.235	0.047	0.138	0.321	<0.001
Perceived opportunity → Intention → Behavioral regulation	0.039	0.012	0.020	0.070	<0.001
Action maintenance					
Behavioral regulation → Habit	0.190	0.060	0.073	0.311	0.002
Behavioral regulation → Identify	0.502	0.043	0.419	0.587	<0.001
Habit → Leisure activity	0.131	0.043	0.046	0.214	0.004
Identify → Leisure activity	0.185	0.048	0.086	0.277	<0.001
Behavioral regulation → Habit → Leisure activity	0.602	0.307	0.158	1.433	0.003
Behavioral regulation → Identify → Leisure activity	2.252	0.688	1.040	3.836	<0.001
Behavioral regulation → Habit/Identify → Leisure activity	2.854	0.738	1.618	4.587	<0.001

## 4 Discussion

The aim of this study was to examine the potential pathway from intention formation to leisure-time physical activity maintenance in older adults. We found a substantial intention-action gap in leisure-time physical activity behavior among community-dwelling older adults. Structural equation modeling revealed that perceived opportunity not only directly influenced the formation of physical activity intention but also, together with intention and affective attitude, impacted behavioral regulation. Furthermore, behavioral regulation positively influenced the leisure-time physical activity behavior of older adults by fostering habit formation and identity affirmation. These findings highlight the importance of perceived opportunity and affective attitude in forming intentions and initiating physical activity, as well as the importance of habits and identity affirmation in maintaining leisure-time physical activity. They provide meaningful evidence for developing targeted interventions to promote physical activity in older adults.

In this study, we found a significant intention-action gap (81.9%) in physical activity behavior. It was much higher than the general adult population (de Bruijn, 2011; Rhodes et al., 2008) (37.9 to 63.8%), and university students (de Bruijn et al., 2012; Rhodes et al., 2012) (42.2 to 55.8%). The great intention-action gap in older adults might be explained by the fact that they usually have higher intentions but poorer actions compared to other groups. Older adults usually experience more urgent health threats from age-related degeneration and chronic diseases (Li et al., 2025), which would prompt them to engage in physical activity for the potential health benefits. Furthermore, older adults have relatively frequent interactions with medical staff who would encourage they to exercise regularly for disease management and health promotion. It can act as an external factor leading to higher intentions for physical activity. However, during the intention translation process, older adults may encounter more barriers, such as limited physical functions, lack of professional knowledge and skills, safety concerns, and excessive protection (Meredith et al., 2023), which may prevent them from translating intentions to actions, creating a large intention-action gap.

To explore the potential pathway linking intention to action among older adults, we performed structural equation modeling. During the intention formation process, we found that perceived opportunity was the only factor related to leisure-time physical activity intention. This finding was in line with previous studies in older adults (Jiao et al., 2022). All together, they highlight the importance of providing older adults with opportunities for leisure-time physical activity, including creating physical and social environment that encourages leisure-time physical activity. Based on previous evidence, favorable environmental conditions, such as safe and convenient facilities and equipment as well as sufficient social support from peers or families, could motivate older adults to form intentions and engage in leisure-time physical activity (Meredith et al., 2023)^,^. In future research, perceived opportunity may serve as a key target for leisure-time physical activity promotion programs among older adults.

It is worth noting that we did not find significant associations of leisure-time physical activity intention with other antecedent variables, including affective attitude, instrumental attitude, and perceived capability. Several reasons may explain the null finding. First, ceiling effect could attenuate associations. A ceiling effect is considered present when more than 15% of participants achieve the maximum possible score on a measure (Terwee et al., 2007). In the current study, 61.3 and 43.9% of respondents reported the maximum score for affective attitude and instrumental attitude, respectively. Furthermore, most of the participants in our study reported high perceived capability score, which might also limit the predictive power of perceived capability for intention formation (Serrano-del-Rosal et al., 2013). Given that common physical activities, such as walking, do not require complex capacities, it is likely that perceived capability may not always correlate with physical activity intention, as previous evidence indicates (Eek et al., 2021). A majority of the participants reported the highest possible scores for affective attitude, instrumental attitude, and perceived capability, suggesting that many older adults already recognize the benefits of physical activity, perceive it positively, and feel capable of engaging in it. Therefore, future interventions should prioritize enhancing perceived opportunities for physical activity over modifying internal cognitions. Such efforts include improving access to age-friendly facilities, strengthening social support, and reducing environmental barriers.

During the formation to adoption process, perceived opportunity was not only directly related to behavioral regulation but also indirectly via intention. It has been widely recognized that intention is the premise for many behaviors. Individuals with higher levels of intention are more likely to engage in actual leisure-time physical activity behavior, particularly in favorable environmental conditions (da Silva et al., 2018), as intention could empower individuals to regulate their behavior. In addition, we found positive affective attitude was associated with better behavioral regulation. This finding is not surprising given that enjoyable emotional experiences from leisure-time physical activity could act as a reinforcer and strengthen behavioral regulations (Marcus et al., 2000). Therefore, elevating individual intention level, creating favorable environmental conditions, and improving affective experiences are all required for initiating leisure-time physical activity behavior among older adults. Meanwhile, it is equally important to maintain leisure-time physical activity behavior after initiating this behavior.

During the process from action adoption to action maintenance, behavioral regulation indirectly influenced leisure-time physical activity via habit and identity. Behavioral regulation, through self-control to overcome barriers, helps maintain the continuity of leisure-time physical activity by enabling the repeated practice that eventually forms habit (Dominguez, 2016). For older adults, cultivating a habit is crucial for maintaining their leisure-time physical activity behavior (Cepni et al., 2024). Additionally, behavioral regulation facilitates the gradual internalization of identity labels among older adults through promoting repeated practice, cognitive adjustments, and social interactions (Strachan et al., 2015). Once this identity is established, regular physical activity becomes an integral part of the individual’s self-concept structure, constituting an important component of their identity (Brassington et al., 2002). The establishment of such an identity could enhance the intrinsic motivation, thereby increasing the likelihood to maintain long-term and stable leisure-time physical activity. Interestingly, there was no direct association between behavioral regulation and leisure-time physical activity in this study. This null finding may be explained by the measurement. Because the behavioral regulation measures reflect current regulatory efforts, while the PA outcome (PASE) captures activity over the past month. This misalignment in the measurement window may attenuate the association. These findings suggest that it is necessary to design and implement appropriate and effective intervention measures targeting the exercise habits and identity of older adults, aiming to encourage them to engage in leisure-time physical activity both long-term and sufficiently.

### 4.1 Strengths and limitations

A major strength of this study was that it explored the pathway from intention to action for leisure-time physical activity among community-dwelling older adult, guided by the M-PAC framework. However, there are some limitations to this study. First, the cross-sectional design precluded us from drawing causal inferences. Although we found several significant pathways from intention formation to maintenance, their causal relationships need to be confirmed by longitudinal or interventional studies. Second, our sample was recruited from Shanghai, a city where physical activity related culture, policies and facilities are relatively more developed compared with other smaller or less economically developed cities. Thus, study findings cannot be generalized to populations from such locations. Additionally, measurement of key variables relied on self-report, which may be subject to recall bias, particularly among this group of older adults. Furthermore, due to the design constraints of the PASE scale, our MVPA calculation exclusively covers leisure-time physical activity, omitting household and occupational activities. Finally, physical activity intention was assessed using a single-item measure, asking about the intended frequency (days per week). Although this approach has been widely used, it may inherently lack the reliability of multi-item scales. Relatedly, actual behavior was evaluated based on activity duration and intensity. This measurement inconsistency may have resulted in an overestimation of the observed intention-action gap. Thus, this finding should be interpreted with caution. Based on the above, more studies are needed to confirm findings from this study. Such studies should consider using objective measures for physical activity and include a more representative sample.

### 4.2 Implications

Despite the above limitations, this study has important implications for clinical practice and future research. In view of the significant intention-action gap in physical activity, healthcare professionals working in the community should move beyond merely focusing on intention formation or exercise prescription. Emphasize should be prioritized to promote the translation from intention to actual action. Healthcare professionals and researchers are encouraged to offer more targeted strategies based on the three stages of physical activity for older adults. At the stage of intention formation, enhancing perceived opportunity is critical to consolidate their motivation and elevate their intention for leisure-time physical activity. It could be achieved by optimizing leisure-time physical activity environment, providing convenient and safe infrastructure, and organizing community activities. For example, community programs could establish structured group-based exercise sessions in local centers and provide low-cost supervised physical activity programs tailored for older adults (Hua et al., 2025). At the stage of action adoption, in addition to possessing a favorable perception of opportunity, positive affective attitude plays a critical role. Thus, how to reduce or overcome practical barriers, such as time conflicts, transportation difficulties, or physical discomfort, should be addressed at this stage. Specific practical support could include providing transportation to exercise facilities, developing indoor alternatives for bad weather, and ensuring safety through supervised group activities (Coletta et al., 2025). Meanwhile, emotional experiences during physical activity should not be overlooked. Strategies, such as building social connections through group activities and providing timely feedback and encouragement, can promote older adults’ adherence to exercise (Arkkukangas et al., 2025). All of the above are essential to successful behavioral regulation and to facilitate a smooth transition from intention to action. Importantly, perceived opportunity may require different intervention approaches across stages. During intention formation, interventions should focus on increasing awareness of available resources and strengthening perceptions that exercise opportunities are accessible and feasible. During action adoption, interventions should emphasize reducing practical barriers that hinder the translation of intention into action. Collectively, stage-specific interventions are necessary to maximize the capacity of perceived opportunity throughout the intention-action process. Finally, at the stage of action maintenance, the core task is to help older adults internalize physical activity as a stable habit and reinforce their identity recognition. For example, digital interventions can support habit formation through behavioral repetition and self-monitoring, while simultaneously cultivating a movement mindset and reinforcing older adults’ identity as active individuals (Kim et al., 2025).

## 5 Conclusion

Guided by the M-PAC framework, this study explored the potential pathway from intention formation to action control of leisure-time physical activity in a sample of older adults. It revealed a significant intention-action gap for physical activity and highlighted the potential importance of improving perceived opportunity and affective attitude in promoting intention formation and facilitating intention translation. It also confirmed the mediating role of habit and identity in the relation between behavioral regulation and leisure-time physical activity maintenance. The above findings provided theoretical references and practical evidence for the design of targeted interventional programs for leisure-time physical activity. In the future, personalized strategies are warranted for older adults at different behavioral stages to promote leisure-time physical activity.

## Data Availability

The raw data supporting the conclusions of this article will be made available by the authors, without undue reservation.
