# Supplementary material for: Physical activity behavior pathway from intention to action in older adults: a structural equation model based on the multi-process action control framework
Source: Front Psychol. 2026 Jul 1;17:1786169. doi: 10.3389/fpsyg.2026.1786169 (PMC13368338; doi:10.3389/fpsyg.2026.1786169)
Supplement: Supplementary file 1 [file Table_1.DOCX]

**Supplementary file**

Table S1 The path results of the hypothetical model

| Path | Total effect | | | Direct effect | | | Indirect effect | | |
| --- | --- | --- | --- | --- | --- | --- | --- | --- | --- |
|  | β (*95% CI*) | *SE* | *p* | β (*95% CI*) | *SE* | *p* | β (*95% CI*) | *SE* | *p* |
| Affective attitude → Behavioral regulation | 0.150 [0.057, 0.238] | 0.046 | **0.003** | 0.125 [0.034, 0.209] | 0.044 | **0.008** | 0.025 [-0.008, 0.070] | 0.019 | 0.122 |
| Perceived opportunity → Behavioral regulation | 0.241 [0.124, 0.358] | 0.059 | **<0.001** | 0.176 [0.066, 0.287] | 0.055 | **0.002** | 0.065 [0.027, 0.117] | 0.023 | **0.001** |
| Behavioral regulation → Leisure-time physical activity | 0.121 [0.029, 0.222] | 0.050 | **0.006** | 0.004 [-0.124, 0.131] | 0.066 | 0.996 | 0.117 [0.056, 0.197] | 0.035 | **0.001** |
| Affective attitude → Intention | 0.109 [-0.044, 0.274] | 0.080 | 0.155 | 0.109 [-0.044, 0.274] | 0.080 | 0.155 | - | - | - |
| Instrumental attitude → Intention | 0.014 [-0.120, 0.148] | 0.070 | 0.824 | 0.014 [-0.120, 0.148] | 0.070 | 0.824 | - | - | - |
| Perceived capability → Intention | -0.080 [-0.220, 0.062] | 0.072 | 0.268 | -0.080 [-0.220, 0.062] | 0.072 | 0.268 | - | - | - |
| Perceived opportunity → Intention | 0.279 [0.107, 0.443] | 0.085 | **0.002** | 0.279 [0.107, 0.443] | 0.085 | **0.002** | - | - | - |
| Intention → Behavioral regulation | 0.234 [0.139, 0.320] | 0.046 | **<0.001** | 0.234 [0.139, 0.320] | 0.046 | **<0.001** | - | - | - |
| Behavioral regulation → Identity | 0.503 [0.419, 0.589] | 0.043 | **<0.001** | 0.503 [0.419, 0.589] | 0.043 | **<0.001** | - | - | - |
| Behavioral regulation → Habit | 0.191 [0.073, 0.313] | 0.060 | **0.002** | 0.191 [0.073, 0.313] | 0.060 | **0.002** | - | - | - |
| Habit → Leisure-time physical activity | 0.131 [0.048, 0.215] | 0.042 | **0.003** | 0.131 [0.048, 0.215] | 0.042 | **0.003** | - | - | - |
| Identity → Leisure-time physical activity | 0.183 [0.055, 0.307] | 0.063 | **0.005** | 0.183 [0.055, 0.307] | 0.063 | **0.005** | - | - | - |
| Instrumental attitude → Behavioral regulation | 0.003 [-0.029, 0.038] | 0.016 | 0.805 | - | - | - | 0.003 [-0.029, 0.038] | 0.016 | 0.805 |
| Perceived capability → Behavioral regulation | -0.019 [-0.055, 0.013] | 0.017 | 0.225 | - | - | - | -0.019 [-0.055, 0.013] | 0.017 | 0.225 |
| Affective attitude → Identity | 0.075 [0.030, 0.128] | 0.025 | **0.002** | - | - | - | 0.075 [0.030, 0.128] | 0.025 | **0.002** |
| Instrumental attitude → Identity | 0.002 [-0.014, 0.020] | 0.008 | 0.802 | - | - | - | 0.002 [-0.014, 0.020] | 0.008 | 0.802 |
| Perceived capability → Identity | -0.009 [-0.029, 0.006] | 0.009 | 0.205 | - | - | - | -0.009 [-0.029, 0.006] | 0.009 | 0.205 |
| Perceived opportunity → Identity | 0.121 [0.062, 0.195] | 0.034 | **<0.001** | - | - | - | 0.121 [0.062, 0.195] | 0.034 | **<0.001** |
| Intention → Identity | 0.117 [0.066, 0.173] | 0.027 | **<0.001** | - | - | - | 0.117 [0.066, 0.173] | 0.027 | **<0.001** |
| Affective attitude → Habit | 0.029 [0.008, 0.066] | 0.014 | **0.002** | - | - | - | 0.029 [0.008, 0.066] | 0.014 | **0.002** |
| Instrumental attitude → Habit | 0.001 [-0.005, 0.009] | 0.003 | 0.749 | - | - | - | 0.001 [-0.005, 0.009] | 0.003 | 0.749 |
| Perceived capability → Habit | -0.004 [-0.014, 0.002] | 0.004 | 0.146 | - | - | - | -0.004 [-0.014, 0.002] | 0.004 | 0.146 |
| Perceived opportunity → Habit | 0.046 [0.015, 0.098] | 0.020 | **0.001** | - | - | - | 0.046 [0.015, 0.098] | 0.020 | **0.001** |
| Intention → Habit | 0.045 [0.015, 0.088] | 0.018 | **0.001** | - | - | - | 0.045 [0.015, 0.088] | 0.018 | **0.001** |
| Affective attitude → Leisure-time physical activity | 0.018 [0.004, 0.043] | 0.009 | **0.004** | - | - | - | 0.018 [0.004, 0.043] | 0.009 | **0.004** |
| Instrumental attitude → Leisure-time physical activity | 0.000 [-0.003, 0.006] | 0.002 | 0.729 | - | - | - | 0.000 [-0.003, 0.006] | 0.002 | 0.729 |
| Perceived capability → Leisure-time physical activity | -0.002 [-0.010, 0.001] | 0.002 | 0.143 | - | - | - | -0.002 [-0.010, 0.001] | 0.002 | 0.143 |
| Perceived opportunity → Leisure-time physical activity | 0.029 [0.007, 0.068] | 0.007 | **0.003** | - | - | - | 0.029 [0.007, 0.068] | 0.007 | **0.003** |
| Intention → Leisure-time physical activity | 0.028 [0.007, 0.061] | 0.014 | **0.004** | - | - | - | 0.028 [0.007, 0.061] | 0.014 | **0.004** |

Table S2 Convergence validity and composite Reliability test

| **Constructs** | **Items** | **Estimate** | **S.E.** | **C.R.** | **λ** | **AVE** | **CR** |
| --- | --- | --- | --- | --- | --- | --- | --- |
| Affective Attitude | AA 1 | 1.000 | -- | -- | 0.997 | 0.991 | 0.997 |
|  | AA 2 | 0.998 | 0.008 | 126.607 | 0.992 |  |  |
|  | AA 3 | 1.008 | 0.006 | 169.887 | 0.997 |  |  |
| Perceived Opportunity | PO 1 | 1.000 | -- | -- | 0.895 | 0.739 | 0.895 |
|  | PO 2 | 0.964 | 0.063 | 15.301 | 0.863 |  |  |
|  | PO 3 | 0.849 | 0.063 | 13.527 | 0.817 |  |  |
| Behavioral Regulation | BR 6 | 1.000 | -- | -- | 0.719 | 0.698 | 0.933 |
|  | BR 5 | 1.129 | 0.082 | 13.792 | 0.736 |  |  |
|  | BR 4 | 1.092 | 0.069 | 15.727 | 0.836 |  |  |
|  | BR 3 | 1.304 | 0.076 | 17.221 | 0.914 |  |  |
|  | BR 2 | 1.267 | 0.074 | 17.146 | 0.910 |  |  |
|  | BR 1 | 1.111 | 0.070 | 15.927 | 0.847 |  |  |
| Habit | HB 1 | 1.000 | -- | -- | 0.973 | 0.941 | 0.985 |
|  | HB 2 | 0.989 | 0.020 | 50.687 | 0.962 |  |  |
|  | HB 3 | 1.009 | 0.015 | 66.167 | 0.989 |  |  |
|  | HB 4 | 0.992 | 0.020 | 49.158 | 0.958 |  |  |
| Identity | ID 4 | 1.000 | -- | -- | 0.656 | 0.764 | 0.927 |
|  | ID 3 | 1.443 | 0.093 | 15.524 | 0.958 |  |  |
|  | ID 2 | 1.406 | 0.092 | 15.319 | 0.938 |  |  |
|  | ID 1 | 1.337 | 0.092 | 14.509 | 0.874 |  |  |

Table S3：Sensitivity analysis based on multiple imputation: Standardized path coefficients (β) with 95% confidence intervals

| Path | Listwise Deletion | Multiple Imputation |
| --- | --- | --- |
|  | β [95% CI] | β [95% CI] |
| **Intention formation** |  |  |
| Perceived opportunity → Intention | 0.289 [0.164, 0.413] | 0.366 [0.277, 0.454] |
| **Action adoption** |  |  |
| Affective attitude → Behavioral regulation | 0.128 [0.037, 0.210] | 0.124 [0.022, 0.226] |
| Perceived opportunity → Behavioral regulation | 0.165 [0.059, 0.278] | 0.078 [-0.032, 0.187] |
| Intention → Behavioral regulation | 0.235 [0.138, 0.321] | 0.284 [0.184, 0.384] |
| **Action maintenance** |  |  |
| Behavioral regulation → Habit | 0.190 [0.073, 0.311] | 0.162 [0.062, 0.262] |
| Behavioral regulation → Identity | 0.502 [0.419, 0.587] | 0.482 [0.403, 0.561] |
| Habit → Leisure activity | 0.131 [0.046, 0.214] | 0.163 [0.066, 0.261] |
| Identity → Leisure activity | 0.185 [0.086, 0.277] | 0.254 [0.158, 0.349] |

*Interpretation: All paths show consistent direction and magnitude across methods. The path “Perceived opportunity → Behavioral regulation” is significant in CC (CI does not include 0) but becomes non‑significant in MI (CI includes 0); however, its effect size is small (β=0.078) and the overall pattern of results remains unchanged, supporting the robustness of the conclusions.
